# Sulfur Supplementation Potentiates the Formation of Meat Aroma Compounds in Thermally Treated Chicken Carcass Hydrolysate

**DOI:** 10.1111/1750-3841.70564

**Published:** 2025-09-14

**Authors:** Xing Zhang, Sidi Ma, Shao‐Quan Liu

**Affiliations:** ^1^ Department of Food Science and Technology National University of Singapore Singapore Singapore; ^2^ National University of Singapore (Suzhou) Research Institute Suzhou Jiangsu China

**Keywords:** chicken, gas chromatography (GC)–ion mobility spectrometry (IMS), gas chromatography–mass spectrometry (GC–MS), Maillard reaction, S‐containing substances

## Abstract

Chicken carcass is a side stream of poultry processing and can be transformed into chicken sauce via hydrolysis and heat treatment. However, the sauce has weaker meaty aroma intensity due to deficiency of sulfur‐containing (S‐containing) amino acids. In this study, three different S‐containing precursors (cysteine, onion, and djenkol bean) were selected for supplementation into chicken carcass hydrolysate to boost the generation of meaty aroma compounds. Cysteine was found to promote the formation of key meaty volatiles like 2‐methyl‐3‐furanthiol and 2‐furfurylthiol. Supplementation of onion or djenkol beans increased the formation of furfuryl methyl sulfide with fresh garlic or onion character aroma. The stable djenkolic acid in djenkol beans likely hindered their effectiveness as a precursor to volatile sulfur compounds. Headspace‐solid‐phase microextraction–gas chromatography–mass spectrometry (HS‐SPME–GC–MS) and HS–GC–ion mobility spectrometry (IMS) detected a total of 60 and 49 volatiles, respectively, notably heat‐generated aroma‐active compounds 1‐octen‐3‐ol, hexanal, and benzaldehyde. This study presents a promising approach to transforming low‐value poultry side streams into flavorful condiments.

## 1 Introduction

Chicken carcasses, as a sidestream of poultry processing, present an opportunity for creating value‐added products such as protein hydrolysates and polyunsaturated fatty acids (Marculescu and Stan 2011; Zhang and Liu 2024). Various methods can be employed to process chicken carcasses, including extraction, enzymolysis, fermentation, heating, and/or combinations thereof; among them, thermal treatment is commonly adopted due to its simplicity and effectiveness. One noteworthy aspect of thermal treatment is its ability to generate Maillard reaction products (MRPs) from chicken bone extract, which may serve as natural flavorings (Chiang et al. 2022; Sun et al. 2014). This highlights the potential for developing flavorful ingredients from chicken carcass sidestreams.

Recently, Zhang and Liu (2024) further explored this potential; they specifically investigated the effects of reducing sugars (xylose, arabinose, glucose, and fructose) on the color, amino acid composition, and volatile compounds in chicken carcass hydrolysates, with the aim of developing chicken‐based flavorings. Their findings indicate that the thermal treatment of the hydrolysates with added xylose generated some volatile flavor compounds; however, there were no typical meaty odorants such as 2‐methyl‐3‐furanthiol (MFT) and 2‐furfurylthiol (FFT) generated (Zhang and Liu 2024).

Numerous odor‐active compounds contribute to meaty aroma in chicken meat, with a significant emphasis on sulfur‐containing (S‐containing) volatiles and furans such as MFT, 2‐furfurylthiol, and 2,5‐dimethyl‐3‐furan. Additionally, lipid‐derived aroma compounds (e.g., 2‐heptanone, 3‐methyl‐2‐butanone, hexanal, and 1‐octen‐3‐ol) also play a vital role in developing the meaty aroma profile (Gonulalan et al. 2004; Sun et al. 2024). Interestingly, many meaty aromas share similar structural features, often characterized by sulfur or oxygen atoms next to two carbon atoms (Sun et al. 2005). This structural similarity is crucial because the presence of sulfur significantly contributes to the savory scents typically associated with cooked meats, as illustrated in Figure S1. These unique structures can be classified into six subclasses based on varying bonding types (Sun et al. 2005).

However, it is important to note that previous research has shown a limited amount of free sulfur (S) compounds in the original chicken carcass hydrolysate. Moreover, there is only a low content of S‐containing free amino acids (FAAs) like cysteine (Cys) and methionine (Met) present (Zhang, Li, et al. 2023). Given this limitation, it is necessary to supplement sulfur sources into chicken carcass hydrolysate before heat treatment.

Cys is commonly utilized in the Maillard reaction to obtain more meaty odorants (Shakoor et al. 2022). In addition to Cys, some natural sulfur sources may also serve as potential precursors to typical meaty volatile compounds. For instance, *S*‐alk(en)yl‐l‐cysteine sulfoxides (ACSOs, alliin, Figure S2a) are distinct S‐containing secondary components found in *Allium* species such as onions, shallots, and garlic, contributing to the aroma and pungency of these plants (Bloem et al. 2005; Sagar et al. 2022).

Another interesting natural sulfur source candidate is the djenkol bean (*Archidendron jiringa*), a regional delicacy in Indonesia, Malaysia, southern Thailand, and Myanmar. These beans are widely available in markets throughout the year and can be eaten roasted or fried. They resemble large, flattened horse chestnuts and emit a subtle sulfurous aroma when crushed. The seed of djenkol beans contains high moisture (58.55%) and crude protein contents (14.19%), resulting in good physico‐chemical, functional, and cooking properties, which could potentially be the ingredient of new food products and formulations (Sridaran et al. 2012).

Although the djenkol bean contains a limited amount of S‐containing amino acids, it has a significant level of djenkolic acid (CAS RN: 498‐59‐9, C_11_H_23_N_3_S_3_O_6_) in the range of 0.3–1.3 g/100 g wet weight (Barceloux 2009). This compound is a cysteine thioacetal of formaldehyde (Barceloux 2009; Figure S2b). Nevertheless, an excessive consumption of djenkolic acid‐rich foods may cause Djenkolism, a kidney disorder documented in Southeast Asia (Sumitro et al. 2020). High intake leads to the accumulation of acids and crystallization in the kidneys, causing injury (Eiam‐Ong and Sitprija 1998). Recent research found that pretreatment like heating, roasting, and soaking could render the seed safe for human consumption (Boughton et al. 2015). The metabolic pathway of djenkolic acid via cystathionase has been proposed (Greenberg et al. 1964), though the enzyme and reaction mechanisms forming cyclic sulfides remain uncharacterized (Suvachittanont et al. 1996). Notably, djenkolic acid in djenkol bean does not form cyclic sulfur compounds, potentially due to the absence of C–S lyases—enzymes present in garlic, onion (Lancaster et al. 2000), durian (Fischer and Steinhaus 2020), and shiitake mushrooms (Schmidberger and Schieberle 2020). Although C–S lyases in these species are well characterized, their counterparts in stink bean remain poorly understood (Zhang, Batra, et al. 2023). And a previous study showed that heating at high temperatures could facilitate the safe djenkol bean seed for human consumption as well as the aroma compounds (Boughton et al. 2015; Lu, Zhang, et al. 2022; Zhang, Batra, et al. 2023).

Given these properties, both onion and djenkol bean were selected for this study with the intent to generate desired meaty odorants.

Thus, the primary aim of this study was to investigate the effects of sulfur source supplementation, specifically Cys, onion, and djenkol bean, on the formation of meaty odorants in thermally treated chicken carcass hydrolysate. This exploration built upon our previous research (Zhang, Li, et al. 2023; Zhang and Liu 2024) and seeks to identify a low‐cost sulfur alternative to cysteine.

## 2 Materials and Methods

### 2.1 Preparation of Chicken Carcass Hydrolysate

Fresh Kampong chicken carcasses were sourced from Aw's Market Fresh Anxin in Singapore. The protease utilized was MEAP (mixed enzymes PB02 for animal proteolysis) from Nanning Pangbo Biological Engineering Co. Ltd. (Nanning, China). The hydrolysis conditions were optimized as detailed by Zhang, Li, et al. (2023). The chicken carcasses were rinsed under running water to eliminate residual blood then cut into approximately 1 × 3 × 5 cm^3^ blocks and minced with a hand‐held blender (HR1613/00, Philips, Koninklijke, the Netherlands). All minced carcasses were combined, packed in food‐grade airtight bags, and stored at −20°C until needed. The optimal hydrolysis parameters included a protease/substrate ratio of 3:100 (w/w), 51.2°C, 4 h, and unadjusted pH (6.62 ± 0.05). To stop the enzymatic reaction, the samples were heated in a water bath (SW22, Julabo, Seelbach, Germany) at 85°C for 15 min. The mixture was then filtered through coconut cloth and coffee filters, yielding a clear liquid. The degree of hydrolysis achieved was 45.44%. On the basis of dry weight, the protein recovery was 50.45% ± 2.05%, with an ash content of 7.29% ± 0.01%, crude protein of 67.67% ± 1.55%, and a fat content of 1.25% ± 0.04% (Zhang, Li, et al. 2023).

### 2.2 Pre‐Treatment of Sulfur Sources

In this study, two kinds of sulfur‐supplement solutions prepared in sterilized deionized (DI) water were added into the chicken carcass hydrolysates. To be specific, the fresh djenkol beans were sourced from a local market, and red onion was purchased from NTUC Fairprice Co. Ltd., Singapore. The beans and onions were washed by the running tap water and then were separated from the pods/peels and inner peels of djenkol bean seeds. After that, the yellow djenkol bean seed and lilac onion bulb were cut into 2 × 2 × 2 cm^3^ cubes and minced using a 1000 W Philips blender (HR2767, Philips Investment Co. Ltd., Shanghai, China) at a bean or onion/DI water ratio of 1:1 (w/v) for 40 s. All the homogeneous solutions were kept at −20°C before use. Cysteine in powder form was added as required.

### 2.3 Thermal Treatment of Chicken Carcass Hydrolysate

A 10‐mL portion of fresh chicken carcass hydrolysates was placed in a 50‐mL Duran laboratory bottle (Merck, Darmstadt, Germany) containing 1.5% (w/v) l‐(+)‐xylose and different amounts of l‐cysteine (Sigma‐Aldrich, St. Louis, MO, USA), on the basis of the preliminary test and previous research (Boughton et al. 2015; Xu et al. 2018), onion and djenkol bean solutions as outlined in Table 1, without adjusting the pH. All samples, except for the unheated control, were subjected to heating at 100°C for 1 h in an autoclave (Hirayama MFG Corp., Kasukabe‐Shi, Saitama, Japan). Following autoclaving, the samples were rapidly cooled in ice water (Zhang and Liu 2024).

**TABLE 1:** Heat treatment conditions for chicken carcass hydrolysates supplemented with various sulfur sources.

No.	Sample name	Abbreviation	Cysteine^a^ (g/10 mL)	Onion^a^ (g/10 mL)	Djenkol bean^a^ (g/10 mL)	Heat treatment
0	Unheated/Heated‐Control^b^	Un‐Control	0	0	0	N.A.
1		H‐Control	0	0	0	Yes
2	Unheated/Heated‐Cysteine^a^	Un‐Cys‐04	0.04	0	0	N.A.
3		Un‐Cys‐08	0.08	0	0	N.A.
4		Un‐Cys‐12	0.12	0	0	N.A.
5		H‐Cys‐04	0.04	0	0	Yes
6		H‐Cys‐08	0.08	0	0	Yes
7		H‐Cys‐12	0.12	0	0	Yes
8	Unheated/Heated‐ Onion^c^	Un‐Oni‐04	0	0.4	0	N.A.
9		Un‐Oni‐08	0	0.8	0	N.A.
10		Un‐Oni‐12	0	1.2	0	N.A.
11		H‐Oni‐04	0	0.4	0	Yes
12		H‐Oni‐08	0	0.8	0	Yes
13		H‐Oni‐12	0	1.2	0	Yes
14	Unheated/Heated‐Djenkol bean	Un‐Djk‐04	0	0	0.4	N.A.
15		Un‐Djk‐08	0	0	0.8	N.A.
16		Un‐Djk‐12	0	0	1.2	N.A.
17		H‐Djk‐04	0	0	0.4	Yes
18		H‐Djk‐08	0	0	0.8	Yes
19		H‐Djk‐12	0	0	1.2	Yes

^a^ l‐cysteine powder; onion solution; djenkol bean solution.

^b^ Unheated blank was prepared and incubated at room temperature (25°C) for 1 h; heated treatment were conducted at 100°C for 1 h. All the samples were added 1.5% (w/v) xylose, except heated blank was prepared without xylose.

^c^ There were three replications of each treatment for data acquisitions (*n* = 3).

### 2.4 Color and pH Measurement

Color measurement was conducted using a protocol described elsewhere (Zhang and Liu 2024). A reflectance spectrometer (CM‐3500 d; Konica Minolta Sensing Inc., Osaka, Japan) equipped with D65 illumination and a 10° observation angle was utilized to assess color changes. The three‐color distribution parameters measured were *a* ^*^ (red > 0, green = 0), *b* ^*^ (yellow > 0, blue = 0), and *L* ^*^ (lightness, with black = 0 and white = 100). The color difference (∆*E*) was calculated using the *L* ^*^, a*, and *b* ^*^ values obtained from the unheated control samples, according to the following equation:

(1) $$\Delta E=\sqrt{{\left(L_{t}-L_{0}\right)}^{2}+{\left(a_{t}-a_{0}\right)}^{2}+{\left(b_{t}-b_{0}\right)}^{2}}$$

The pH values of each sample were measured using a SevenCompact pH meter (Mettler‐Toledo 135 International Inc., Columbus, OH, USA).

### 2.5 Analysis of Volatile Compounds

#### 2.5.1 Headspace‐Solid‐Phase Microextraction–Gas Chromatography–Mass Spectrometry (HS‐SPME–GC–MS)/Flame Ionization Detection (FID)

The analysis of volatiles in thermally treated chicken carcass hydrolysates followed a modified protocol of Vong and Liu (2017). Before extraction, the samples were thawed in a water bath at 60°C for 5 min. Volatiles were extracted using headspace‐solid‐phase microextraction (HS‐SPME) combined with gas chromatography–mass spectrometry (GC–MS) and quantified by FID (Agilent 5975C, Santa Clara, CA, USA). A 5‐mL aliquot of the samples and 2 g of sodium chloride were added in a 20‐mL glass vial with a polytetrafluoroethylene (PTFE) septum. Subsequently, 3.33 µg of 99.5% (±)‐2‐octanol (not detected in samples) was added into a 5 mL sample as an internal standard (Sigma‐Aldrich, St. Louis, MO, USA).

An 85‐µm carboxen/polydimethylsiloxane SPME fiber (CAR/PDMS, Supelco, Bellefonte, PA, USA) was used to extract volatiles from the headspace at 60°C for 30 min with agitation at 250 rpm using a Combi PAL autosampler (CTC Analytics, Zwingen, Switzerland). The extracted compounds were separated on a nitroterephthalic‐acid‐modified polyethylene glycol (DB‐FFAP) capillary column (60 m length, 0.25 mm i.d., 0.25 µm film thickness, Agilent, Santa Clara, CA, USA) with helium as the carrier gas at 1.2 mL/min. Separation conditions were adapted from Gao et al. (2016), starting at an oven temperature of 40°C for 3 min, then ramping up to 90°C at a rate of 5°C per minute, and finally to 230°C at 10°C per minute, holding for 7 min. Compound identification was based on mass spectral comparisons with NIST 23.0 and Wiley 275 MS libraries, as well as their linear retention index (LRI) values calculated using retention times and a series of alkane standards.

The volatiles were semi‐quantitated by using 2‐octanol (not detected in samples) as an internal standard. LRI was calculated using the following equation:

(2) $$\mathrm{LRI}=100n+100\left(t_{x}-t_{n}\right)/\left(t_{n+1}-t_{n}\right),$$

where *t_x_*, *t_n_*, and *t_n_*_+1_ are the GC–MS retention times of compound *x*, alkane *n*, and alkane *n* + 1 (*t_n_* < *t_x_* < *t_n_*_+1_), respectively.

The content of each volatile was calculated using the following equation:

(3) $$C_{v}=\frac{S_{v}}{S_{a}}\times C_{a},$$

where *S_a_* and *S_v_* represent the GC–FID peak area of 2‐octanol and volatile, respectively. *C_a_* represents the concentration of 2‐octanol.

Odor activity value (OAV) was calculated as in the following equation:

(4) $$\mathrm{OAV}=\frac{C_{v}}{OT_{v}},$$

where *C_v_* refers to volatile content, and OT*v* represents the odor threshold values of volatiles in water and was obtained according to data available in the literature shown in Table 3.

#### 2.5.2 HS–GC–Ion Mobility Spectrometry (IMS)

The volatile fingerprints of all samples were also analyzed using an HS–GC–IMS system (Flavorspec, Gesellschaft für Analytische Sensorsysteme/GAS mbH, Dortmund, Germany), on the basis of a method adapted from Tian et al. (2023). The GC–IMS analysis was performed by Hanon, Beijing, China. Each sample (1.0 mL) was placed in a 20‐mL headspace vial with a PTFE septum and incubated at 60°C for 15 min with 500 rpm agitation using a Combi PAL autosampler (CTC Analytics). The volatiles were separated on an MXT‐WAX capillary column (30 m × 0.53 mm × 1.0 µm, Restek, PA, USA). After incubation, 500 µL of headspace was automatically injected through a heated syringe set at 85°C. The column was maintained at 60°C, using nitrogen (99.999% purity) as both the carrier gas and drift gas. The separation conditions followed those described by Zhang and Liu (2024). After being eluted in the isothermal mode, the analytes were driven into the ionization chamber before detection by the IMS. Molecules were ionized and then driven to the drift tube, which was operated at 45°C and a drift gas flow rate of 150 mL/min. The GC–IMS data were analyzed by using the software Laboratory Analytical Viewer (LAV, Version 2.2.1, GAS mbH, Dortmund, Germany). Identification of volatiles was achieved by comparing the mass spectra of samples with those of the NIST 2023 and IMS mass spectral databases built into the VOCal 0.4.03 software. Retention indices were calculated by using an *n*‐ketones series (C4–C9) under the same chromatographic conditions as the samples.

### 2.6 Statistical Analysis

All results were obtained from three independent experiments conducted on different dates. One‐ or two‐way analysis of variance (ANOVA) and Duncan's multiple range tests were performed for each data set by using SPSS 27.0 (SPSS Inc., Chicago, IL, USA). The heatmap of volatiles was created on OmicShare Tools (https://www.omicshare.com/tools/home/report/reportheatmap.html). The bar graphs were plotted by GraphPad Prism 8.0. The orthogonal least partial squares‐discriminant analysis (OPLS‐DA) was performed using SIMCA software (version 14.1 Umetrics, Umea, Sweden). The FID peak areas were subjected to missing value estimation and normalization by data scaling, that is, auto‐scaling by mean‐centered and divided by the standard deviation of each variable prior to analysis. The proposed reaction schemes were constructed by using ChemDraw (version 22.00.64‐bit, Waltham, MA, USA).

## 3 Results and Discussion

### 3.1 pH Changes

**FIGURE 1:**
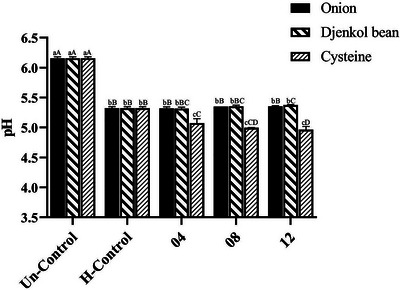
The changes of pH in unheated and heated chicken carcass hydrolysates with S‐supplementations (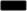
Onion, 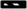
Djenkol bean, 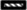
Cysteine).Values within the same dosage on different types of S‐supplementation by the same letters (a, b, c) are not significantly different (*p* > 0.05). Values between the different dosages on the same S‐supplementations followed by the same letters (A, B, C) are not significantly different (*p* > 0.05). *Source*: Sample identities can be found in Table 1.

The pH plays an important role in Maillard reaction. Lower pH (6.5–7.0) results in less nucleophilic amino groups, leading to enhanced reactivity compared to higher pH (8.0–8.5) (Wei et al. 2019). First, a general decrease of pH was found in all heated samples compared with Blk, with Figure 1 showing that pH reduced significantly (*p *< 0.05). Interestingly, there were no significant changes among samples with added onion and djenkol bean. On the other hand, a correlation between pH and the Cys amount was observed after heat treatment, namely, the higher the Cys addition, the greater the decrease in pH, which was in line with the previous research (Zhang et al. 2018). This reduction in pH was likely due to the formation of formic and acetic acids from the breakdown of xylose, FAAs, and peptides in the chicken carcass hydrolysate and the consumption of amino groups. In addition to cysteine, glycine (Gly) has a similar effect on pH reduction. Previous studies showed that the pH value of the reaction system initially decreased with increasing glycine addition, primarily due to the generation of acidic substances through glycine's participation in Maillard reactions and Strecker degradation. However, the pH stabilization observed at higher glycine concentrations was attributed to reaction inhibition under acidic conditions combined with excess amino acid in the system (Hou et al. 2017; Zeng et al. 2012). The relatively stable cyclic 2‐threityl‐thiazolidine‐4‐carboxylic acid (TTCA), rather than the Amadori rearrangement product (ARP) of cysteine (Cys‐Amadori), is primarily formed in the early stage of the Maillard reaction, which circumvents the development of meaty volatiles from cysteine. The conversion from TTCA to ARP was further improved in the range of 7.5–9.5, and enhanced alkaline within this scope would promote this transformation more significantly (Zhai et al. 2020).

### 3.2 Color Changes

The color differences were evaluated to understand color changes in heated chicken carcass hydrolysates. In general, the ∆*E* value of each sample was raised compared with the H‐Blk (9.31 ± 0.46), showing a yellowish transparent color (Table 2, Figure S3). Interestingly, the *L* ^*^ and *b* ^*^ values of Cys addition were increased significantly (*p *< 0.001), but *a* ^*^ was reduced compared to H‐Blk (Table 2). The previous research showed that the addition of Cys could inhibit the browning intensity, which was in line with this study (Zhang et al. 2018). Cys contains a free sulfhydryl group, which can react with reducing sugars to form stable, colorless products first, thereby inhibiting the reaction between sugars and amino acids, resulting in less browning (Chen et al. 2022). Cys might also inhibit the formation of dicarbonyl compounds, which in turn reduces the development of visible colors (Zhai et al. 2019). However, there were no obvious color differences between onion and djenkol bean–added samples (Table 2).

**TABLE 2:** Color changes of unheated and heated chicken carcass hydrolysates with or without supplementation with sulfur sources.

	Color			
Sample name	*L* ^*^	a*	*b* ^*^	∆*E* ^a^
H‐Blk	81.72 ± 0.99	2.96 ± 0.44	49.22 ± 0.46	9.31 ± 0.46
H‐Control	11.54 ± 0.75	33.74 ± 0.35	18.78 ± 0.75	81.11 ± 0.58
H‐Cys‐04	77.23 ± 1.54Aa	10.71 ± 0.46Aa	87.96 ± 0.86Aa	55.38 ± 0.21Aa
H‐Cys‐08	81.72 ± 0.70Ba	8.13 ± 0.37Ba	100.03 ± 0.25Ba	71.06 ± 0.21Ba
H‐Cys‐12	82.11 ± 0.99Ba	7.79 ± 0.75Ba	100.38 ± 1.31Ba	81.15 ± 0.17Ba
H‐Oni‐04	13.72 ± 0.91Ab	34.24 ± 1.03Ab	20.77 ± 0.83Ab	78.87 ± 0.87Ab
H‐Oni‐08	14.53 ± 0.87Ab	35.12 ± 1.33Ab	26.46 ± 0.71Bb	77.19 ± 0.90Ab
H‐Oni‐12	15.23 ± 0.25Ab	37.76 ± 1.20Bb	29.50 ± 1.21Cb	77.22 ± 0.57Ab
H‐Djk‐04	19.47 ± 0.69Ac	33.02 ± 0.64Ab	19.01 ± 1.21Ab	79.76 ± 0.12Ab
H‐Djk‐08	14.43 ± 0.88Bb	34.96 ± 0.98ABb	21.58 ± 1.29Ba	83.30 ± 0.27Bc
H‐Djk‐12	14.33 ± 0.85Bb	35.63 ± 0.23Bb	25.53 ± 1.18Cc	81.95 ± 0.56BCc
Test of significance between effects, *p* Main effect				
Sulfur type	***	***	***	***
Sulfur dosage	N.S.	*	***	*
Sulfur type × dosage	***	***	***	*

*Note*: The color changes between Un‐control and other unheated samples are the same. The values within the same dosage sample on different types of S‐supplement followed by the same letters (a, b, c) are not significantly different (*p* > 0.05). The values between the different dosage samples on the same S‐supplement followed by the same letters (A, B, C) are not significantly different (*p *> 0.05). N.S.: no significant differences (*p >* 0.05).

^a^ All values are the mean ± standard deviation with three replications of each sample treatment (*n* = 3).

*Statistically significant differences (*p ≤* 0.05).

**Statistically significant differences (*p ≤* 0.01).

***Statistically significant differences (*p ≤* 0.001).

### 3.3 Volatile Compounds

#### 3.3.1 GC–MS Analysis

**FIGURE 2:**
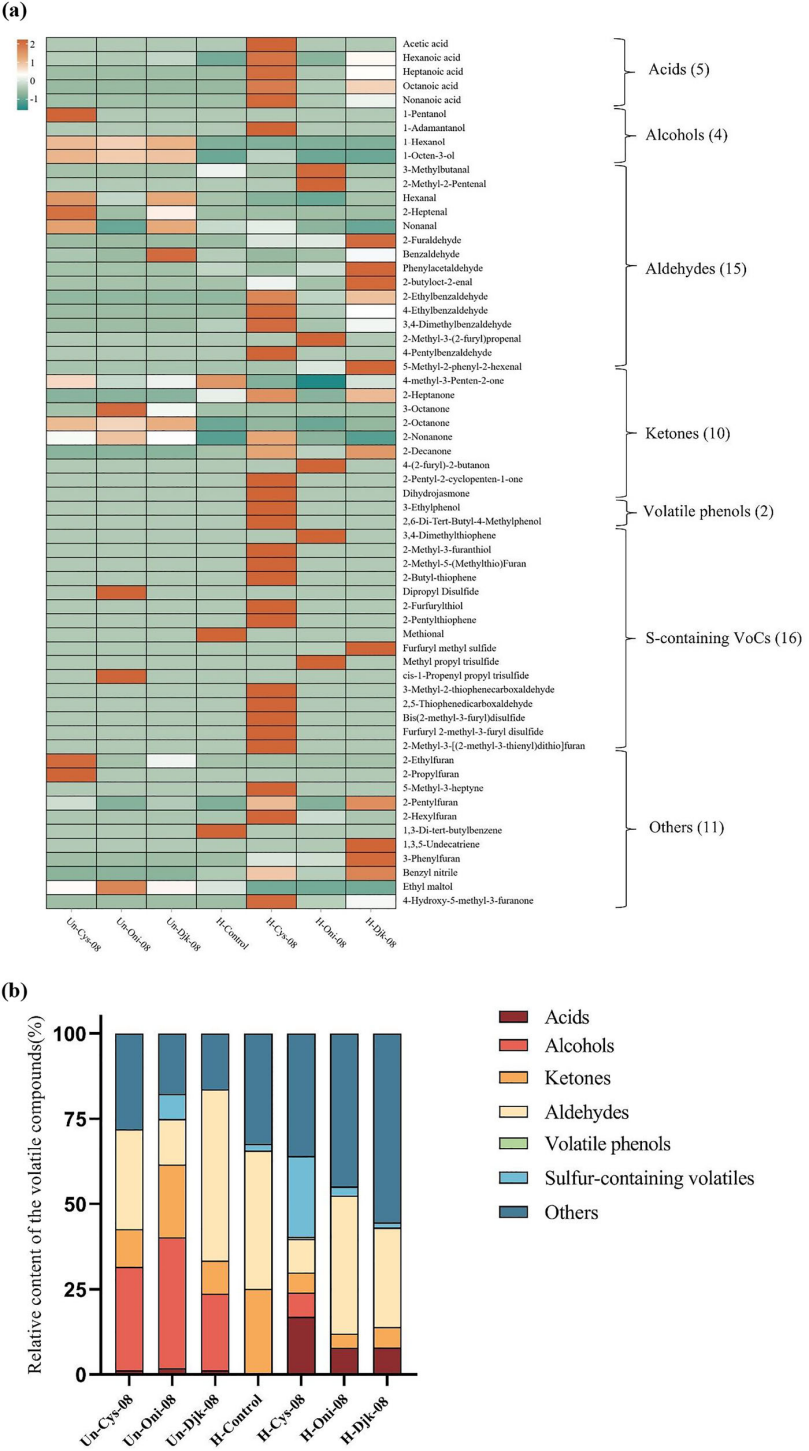
(a) Heatmap of unheated control and heat‐treated chicken carcass hydrolysates with different S‐supplementations by GC–MS/FID. Every square represents a volatile compound. The color of the square would be closer to green if a compound had a lesser concentration; otherwise, it would be closer to red. (b) Relative contents of volatile compounds in chicken carcass hydrolysates with S‐supplementations by GC–MS/FID. Refer to Table 1 for sample identification.

Table S1 shows the volatiles and their relative abundance obtained using HS‐SPME–GC–MS. The heat map in Figure 2a reveals a total of 60 aroma components detected by GC–MS, which can be divided into seven types, including acids (5), alcohols (4), aldehydes (15), ketones (9), volatile phenols (2), S‐containing volatiles (16), and others (9). Furthermore, the relative content of each group is summarized in Figure 2b.

Before heat treatment, alcohols were the dominant compounds in Cys‐Blk‐08 (30.30%) and Oni‐Blk‐08 (38.41%). In contrast, aldehydes made up more than half of the total volatiles in Djk‐Blk‐08 (50.21%). After heat treatment, the “Others” category became the predominant group in all heated samples (H‐Cys‐08, H‐Oni‐08, H‐Djk‐08) except for H‐Control, comprising 35.97%, 44.85%, and 55.41%, respectively. This category contained many volatiles, including heterocyclic compounds like 2‐pentylfuran. Remarkably, the OAV of 2‐pentylfuran increased significantly (*p *< 0.05) after heating, rising from 110 to 244 in H‐Cys‐08 and from 82 to 309 in H‐Djk‐08, whereas onion had no apparent effect on 2‐pentylfuran (Table 3). Furthermore, aldehydes were the second dominant category in H‐Oni‐08 and H‐Djk‐08, representing 40.49% and 29.10%, respectively.

**TABLE 3:** Effect of S‐supplements on volatiles (OAV ≥ 1) in unheated and heated chicken carcass hydrolysates with or without S‐supplements.

Compound identified	RI^a^	Odor threshold (µg/L)	Odor activity value (OAV)						
			Unheated control and heated control			Heated samples with different S‐supplements			
			Un‐Cys‐08	Un‐Oni‐08	Un‐Djk–08	H‐Control	H‐Cys‐08	H‐Oni‐08	H‐Djk‐08
**Alcohol**		—	—	—	—	—	—	—	—
1‐Octen‐3‐ol^b^	1456	1.5	186	164	174	—	59	—	—
**Aldehyde**		—	—	—	—	—	—	—	—
3‐Methylbutanal^b^	927	4.0	—	—	—	4	—	14	—
Hexanal^b^	1084	5.0	127	41	114	28	15	5	28
**Ketone**		—	—	—	—	—	—	—	—
2‐Heptanone^c^	1201	49.0	—	—	—	—	2	—	2
3‐Octanone^b^	1300	21.5	—	5	1	—	—	—	—
2‐Octanone^b^	1291	50.3	3	3	4	—	1	—	1
**Sulfur‐containing volatiles**		—	—	—	—	—	—	—	—
2‐Methyl‐3‐furanthiol (MFT)^b^	1319	0.007	—	—	—	—	22,803	—	—
Dipropyl disulfide^d^	1358	0.9	—	30	—	—	—	—	—
2‐Furfurylthiol (FFT)^b^	1435	0.005	—	—	—	—	42,609	—	—
Methional^d^	1449	0.2	—	—	—	72	—	—	—
bis(2‐Methyl‐3‐furyl) disulfide (MFT–MFT)^b^	2153	0.00002	—	—	—	—	28,987,837	—	—
**Others**									
2‐Pentylfuran^b^	1244	5.8	110	39	82	34	244	37	309

*Note*: “—” indicates that the substance has not been detected.

^a^ Retention index were obtained from NIST Chemistry WebBook, SRD 69 (https://webbook.nist.gov/chemistry/). (https://www.chemicalbook.com), PubChem database (https://pubchem.ncbi.nlm.nih.gov) and Sigma‐Aldrich (https://www.sigmaaldrich.com).

^b^ Odor thresholds: value obtained from Münch and Schieberle (1998).

^c^ Odor thresholds were obtained from Odor Thresholds for Chemicals with Established Occupational Health Standards, 3rd edition, Chemical Book database

^d^ Odor thresholds: value obtained from Leffingwell, D.L. and J.C. Leffingwell, J.C (2003).

Notably, the relative content of S‐containing volatiles was the second highest, followed by the category “Others” in sample H‐Cys‐08; however, there was no S‐containing volatile with OAV ≥ 1 generated in H‐Oni‐08 and H‐Djk‐08, even though there were two compounds with OAV ≤ 1 detected (furfuryl methyl sulfide and methyl propyl trisulfide). Among the S‐containing volatiles, FFT, MFT, and MFT–MFT were the typical meaty‐like aroma compounds generated during the cooking of chicken meat (Jayasena et al. 2013). In H‐Cys‐08, the OAVs of them were extremely high (28,987,837, 42,609, and 22,803, respectively (Table 3)), due to their very low thresholds (Gao et al. 2020; Jayasena et al. 2013). Previous studies also revealed the flavor dilution factors of MFT and FFT in chicken broth were 1024 and 256, respectively, showing their importance as the most vital meaty‐like volatile compounds (Jayasena et al. 2013). According to Zhang, Batra et al. (2023), djenkolic acid structure is stable and does not participate in the Maillard reaction. On the other hand, some of the alliin could be degraded by heating but not converted into some key intermediates for the formation of meaty odorants.

**FIGURE 3:**
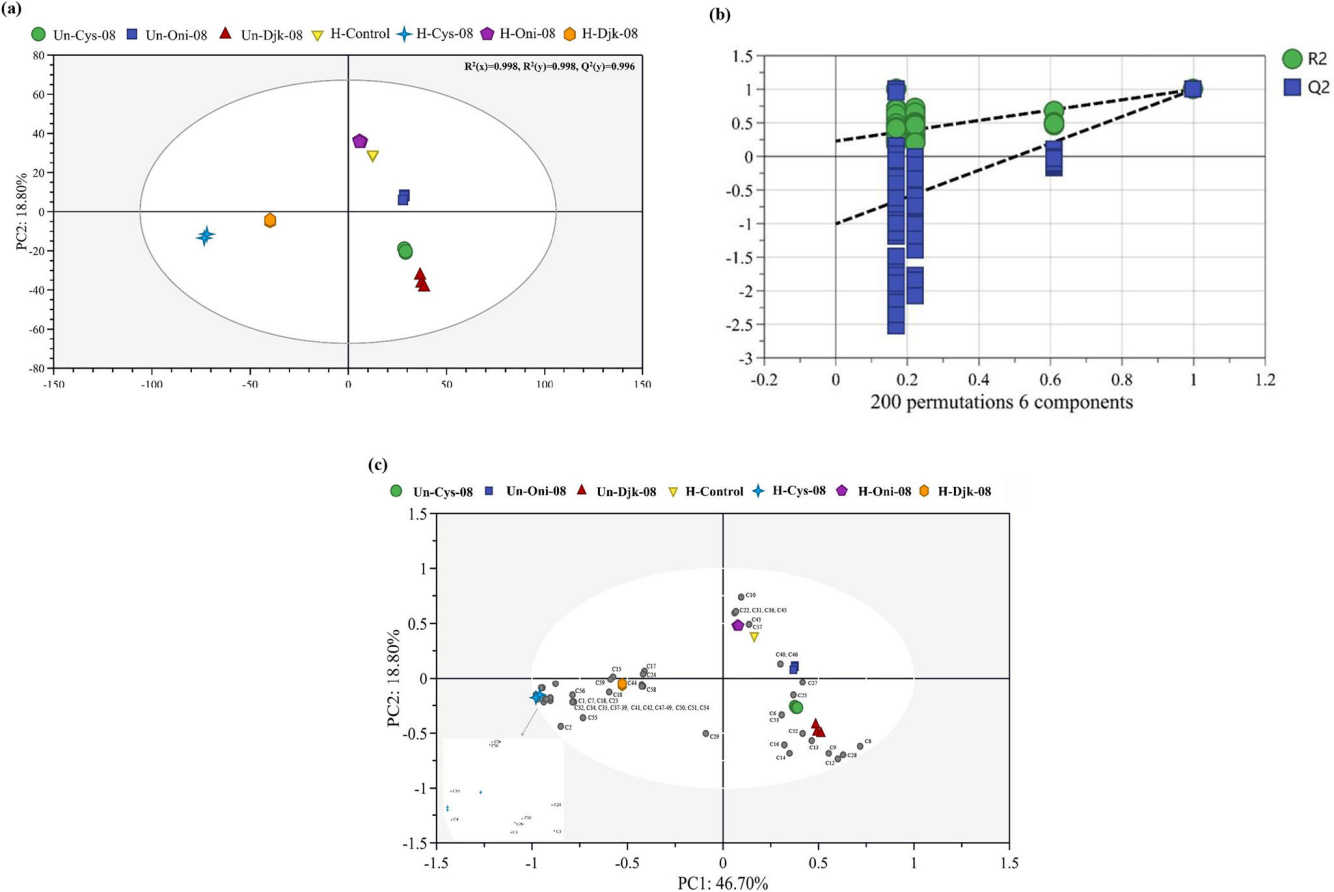
(a) OPLS‐DA score plots of samples by HS–SPME–GC–MS, (b) permutation test results (*n* = 200) of OPLS‐DA mode of GC–MS, and (c) biplot of volatiles produced in samples as described in Table 1; numbers 1–60 refer to the volatiles in Table S1.

Using the 60 volatiles as dependent variables and the different S‐containing sources as independent variables, OPLS‐DA analysis (Figure 3a) effectively distinguishes the different thermally reacted chicken carcass hydrolysates. In this analysis, the fitting indices for the independent variables (*R* ^2^ *x*) are 0.998 and 0.998, respectively, and the model's predictive index (*Q* ^2^) is 0.996. Because *R* ^2^ and *Q* ^2^ exceed 0.95, the model fitting results are considered acceptable (Lu, Liu et al. 2022). Following 200 permutation tests (Figure S4), the intersection of the *Q* ^2^ regression line with the vertical axis is below zero, indicating that the model is not overfitting and is valid. Thus, the results are deemed suitable for the analysis of different thermally reacted chicken carcass hydrolysates.

The outcomes of the OPLS‐DA grouping align well with the classification results obtained from the heat map (Figure 3b). In addition, the components with variable importance of projection (VIP) values greater than one are regarded as distinctive volatiles (Lu, Liu, et al. 2022). As can be observed in the score plot of the OPLS‐DA model of HS‐SPME–GC–MS, the volatiles from all seven samples, including blank and controls, were clearly discriminated. The main characteristic volatile components (VIP > 1) in H‐Cys‐08 were C41 (FFT), C49 (MFT–MFT), and C4 (octanoic acid), which were related to the meaty and fatty aroma (Gao et al. 2020). Moreover, H‐Djk‐08 had a higher relevance to C59 (3‐phenylfuran) and C44 (furfuryl methyl sulfide) as associated with onion character aroma (Zhang, Wang, et al. 2023). In other quadrants, H‐Oni‐08 and H‐Control had higher relevance to C22 (2‐methyl‐3‐(2‐furyl) propenal) and C43 (methional), attributed to cooked potato and meat broth aroma (Li and Liu 2022).

#### 3.3.2 GC–IMS analysis

**FIGURE 4:**
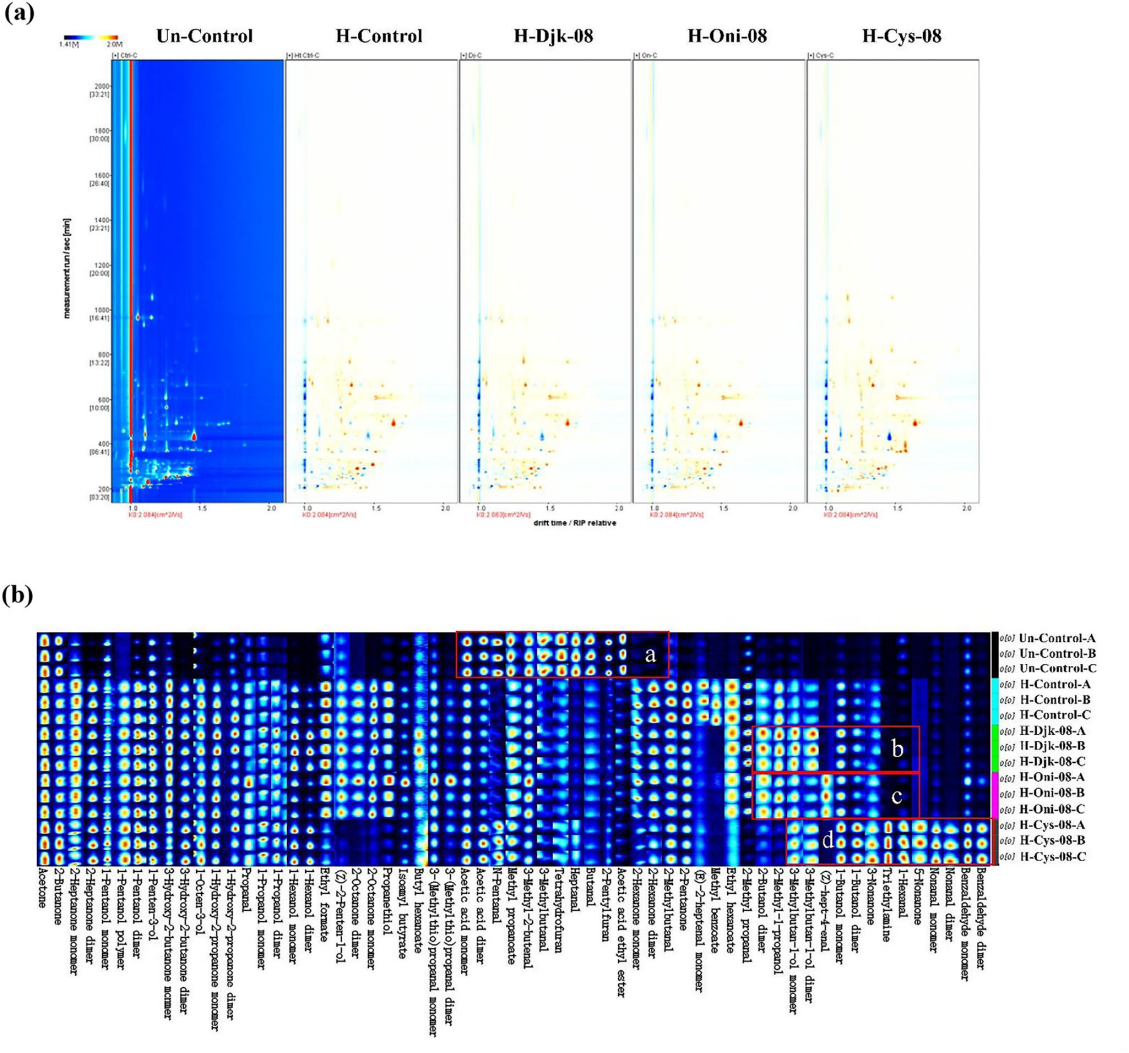
Volatile compounds’ fingerprints of unheated control and heated chicken carcass hydrolysates with S‐supplementations. (a) GC–IMS 2D‐topographic plot, if the compounds are consistent with unheated control, the background was white after deduction; red means that concentration of the compounds was higher than unheated control, and blue means lower. (b) Gallery plot, each row represents one sample, and each column represents volatile, and the stronger the brightness of the square, the higher the concentration of that substance. The letters a, b, c, and d represent the characteristic volatility distinctions for different samples.

Through drift time (*X*‐axis), retention time (*Y*‐axis), and various colors, top views are presented to visualize the volatiles’ differences (Figure 4a). Subsequently, the volatiles were identified by integrating retention indices, retention times, and drift times utilizing data from the IMS Library. The volatiles’ fingerprints clearly show that H‐Cys‐08 differed most markedly from other samples (Figure 4b). Moreover, the right part of this fingerprint indicated that new volatiles were produced after heating with added Cys and onion, rather than djenkol bean.

Table S2 presents that 49 volatiles were identified (except 46 unqualified peaks, dimers, and polymers), including ketones (10 types), acids (1 type), esters (10 types), alcohols (11 types), aldehydes (8 types), S‐containing compounds (2 types), and other substances (2 types). The volatiles in Region a (inside the red box) of Figure 4b show higher concentrations of acetic acid ethyl ester, 2‐pentylfuran, butanal, heptanal, and 3‐methylbutanal in Blk. After heating, all of them were below their detection limits. Besides, 3‐methylbutan‐1‐ol, 2‐methy‐1‐propanol, and 2‐butanol were detected in both H‐Djk‐08 and H‐Oni‐08 (Meng et al. 2021), whereas (*Z*)‐hept‐4‐enal was only found in H‐Oni‐08 (shown in Regions b, c). Benzaldehyde, nonanal, and 1‐hexanal were detected in H‐Cys‐08 only, among which benzaldehyde imparts almond and nutty characters in meat products (Yu et al. 2025).

Only two S‐containing volatiles, methional and propanethiol, were detected by GC–IMS, which is much less than the 16 types identified by GC–MS. Among which, methional, the Strecker aldehyde of methionine known for its cooked potato scent, consistently shows the highest flavor dilution factor in teas such as Chinese Sencha and Keemun black tea (Xiao et al. 2024). Another study revealed that the methionine/glucose‐derived Amadori rearrangement product (MG‐ARP) exhibited a higher reactivity to create methional than the methionine/glucose mixture model, but it produced fewer species and had a lower pyrazine concentration (Deng et al. 2022). Besides, as a type of highly volatile odorants hydrogen sulfide, propanethiol was also found in durian pulp (Li et al. 2017). GC–IMS has gained popularity in the food volatile compounds in recent years due to its many advantages. In contrast to GC–MS, which necessitates elevated temperatures, GC–IMS operates at room temperature. This allows for the direct and time‐efficient detection of volatiles without requiring sample enrichment. GC–IMS demonstrates high sensitivity, capable of detecting substances at parts per billion (ppb) levels, and effectively distinguishes between odorant isomers based on their molecular weight and structure. Additionally, GC–IMS produces visual fingerprint spectra that enhance the clarity of odorant differentiation easily, making the analysis and interpretation processes simpler (Wang et al. 2020). However, the reason why GC–IMS failed to detect the key sulfur volatiles like MFT in this study might be that GC–IMS utilizes soft ionization, primarily generating reactant ions (such as H_3_O⁺(H_2_O)*_n_*). Compounds form ions by reacting with these reactant ions through processes like proton transfer reactions (for compounds with proton affinity higher than water) or other reactions (such as charge transfer and ligand switching). Many volatile sulfur compounds (VSCs) have relatively low proton affinity, approaching or falling below that of the hydronium ion (Wang et al. 2020). This results in inefficient ionization or very low ionization efficiency for these compounds. Consequently, their signals in IMS are extremely weak or even undetectable. Besides, GC–IMS primarily relies on drift time (related to collision cross section) and retention index for compound identification. Currently available, standardized GC–IMS databases containing a large number of sulfur compounds are relatively underdeveloped and less widespread compared to GC–MS libraries. Different compounds with similar collision cross sections may be erroneously assigned. Accurately identifying unknown sulfur compounds presents a greater challenge (Wang et al. 2020; Zhang et al. 2021, 2025). However, limitations, such as the nonlinear detector used in conjunction with GC and the absence of a robust spectral library (e.g., the NIST mass spectral library), present challenges, which demand further advancements to enhance GC–IMS capabilities (Yuan et al. 2024; Huang et al. 2025). Currently, multiple methods such as GC–MS, GC–olfactometry (O)–MS, or GC × GC‐Time of Flight (ToF)–MS appear to be most common and effective (Wang et al. 2020; Zou et al. 2023).

#### 3.3.3 Proposed Reaction Schemes for Formation of Key Aroma Compounds

**FIGURE 5:**
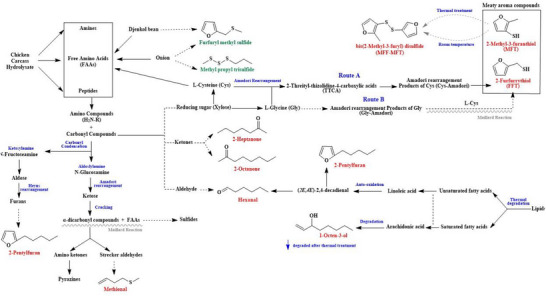
Proposed reaction schemes of formation of key aroma compounds in chicken carcass hydrolysate heated with or without S‐supplementation. *Source*: Adapted from Lu, Zhang, et al. (2022), Ding et al. (2020), Gao et al. (2020), Cao et al. (2017), Hou et al. (2017), and Gong et al. (2016).

Figure 5 summarizes the proposed reaction schemes of formation of these key meaty volatiles, namely, hexanal, 1‐octen‐3‐ol, 2‐pentylfuran, methional, MFT, FFT, and MFT–MFT. Hexanal was deemed as an indicator of lipid oxidation, and it participated in the reaction of cysteine and reducing sugar during the Maillard reaction (Yang et al. 2015). In addition, 1‐octen‐3‐ol is an oxidative product of arachidonic acid and linoleic acid through auto‐oxidation, and its amount is reduced remarkably after heating (Ding et al. 2020), and 2‐pentylfuran is commonly formed from the oxidation of linoleic acid (Li and Liu 2021).

The inclusion of xylose enhanced the production of various “cooked and savory” volatile compounds, including methional and furfuryl methyl sulfide during heat treatment. When xylose is present alongside amino acids or peptides, it can lead to the formation of Amadori compounds under moderate heating conditions, which can subsequently give rise to dicarbonyl compounds (Cao et al. 2017). These dicarbonyl compounds can further react with methionine through Strecker degradation to produce methional and furfuryl methyl sulfide (Zhang et al. 2019).

In a meat‐like Maillard reaction system involving cysteine and reducing sugars like xylose, the initial formation of relatively stable 2‐threityl‐thiazolidine‐4‐carboxylic acids (TTCA) hinders the progression toward meaty volatile compounds’ development. Additionally, these thiazolidines can inhibit browning (Section 3.2). However, with prolonged heating, TTCA can be reversibly converted into the Cys‐Amadori product, a process that can be catalyzed by other amino acids such as glycine (Gly) in an acidic environment (Cao et al. 2017; Gao et al. 2020; Hou et al. 2017). Accordingly, the S‐containing meaty odorants (e.g., MFT and FFT) can be formed through two routes: the degradation of Cys‐Amadori intermediates (Route A, shown in Figure. 5) and/or the interaction of cysteine with the degradation products of Gly‐Amadori intermediates (Route B, shown in Figure. 5). Experimental evidence confirms glycine's catalytic role in accelerating the Maillard reaction between cysteine and reducing sugars, effectively generating meat‐like aroma compounds through a characterized general acid catalysis mechanism (de Roos et al. 2005; Gong et al. 2016). This catalytic enhancement likely facilitates accelerated Cys‐Amadori degradation, thereby promoting H_2_S generation. The liberated H_2_S may subsequently undergo nucleophilic attack on Maillard‐derived chromophoric degradation byproducts, ultimately driving the formation of S‐containing heterocycles (e.g., thiophenes and thiazoles) (Martins et al. 2010; Hou et al. 2017). On the other hand, Gly exhibited catalytic acceleration of the reversible transformation between TTCA and Cys‐Amadori intermediates in the TTCA/glycine system, suggesting a proton‐transfer‐mediated mechanism potentially involving dual acid‐base catalytic sites (de Roos et al. 2005; Gong et al. 2016). However, there is limited information on how glycine competes with cysteine in this initial stage of the Maillard reaction and the resulting formation of intermediate products and aroma‐forming pathways (Gong et al. 2016). MFT–MFT, a potent meaty and roasted odorant with a low‐odor threshold, was detected in this study and elsewhere (Gao et al. 2020). Nonetheless, it is unclear whether MFT–MFT was induced by heat treatment because this odorant can be formed by MFT oxidation during room temperature storage or during GC analysis (Guth et al. 1995).

## 4 Conclusion

This study investigated the impact of three S‐containing substances, cysteine, onion, and djenkol bean, on the generation of meaty aroma compounds heated with chicken carcass hydrolysate. It was found that only Cys significantly reduced pH and inhibited browning, facilitating the production of key meaty odorants such as MFT and FFT. In contrast, the stable djenkolic acid in djenkol bean limited its effectiveness as a precursor, and the fresh garlic/onion‐flavored furfuryl methyl sulfide was formed in both onion and djenkol bean added samples. A total of 60 and 49 volatiles were identified across all samples using HS‐SPME–GC–MS and HS–GC–IMS, respectively, with key aroma‐active substances, including 1‐octen‐3‐ol, hexanal, and benzaldehyde, being linked to lipid oxidation or the Maillard reactions.

Emerging evidence demonstrates that pyridoxal 5′‐phosphate‐dependent lyases—including C–S lyase (EC 4.4.1.8), cystathionine γ‐lyase (EC 4.4.1.1), *S*‐alkylcysteine α,β‐lyase (EC 4.4.1.6), and cystathionase (EC 4.4.1.2)—exhibit catalytic capacity for the α/β‐elimination‐mediated degradation of djenkolic acid (Greenberg et al. 1964; Gmelin et al. 1981). Notably, stink bean–derived C–S lyase further mediates the enzymatic synthesis of cyclic organosulfides (COSs), generating bioactive thiosulfinates such as 1,2,4‐trithiolane (TTL), 1,2,4,6‐tetrathiepane (TTP), and lanthionine (LEN) via djenkolic acid bioconversion (Zhang, Batra, et al. 2023). Future research should focus on optimizing enzyme treatments of djenkol beans to enhance the cleavage of djenkolic acid by C–S lyase based on the optimized condition by previous research (pH = 9, 50°C, etc. Zhang, Batra, et al. 2023), thus improving their utility as active precursors. This study highlights a possible method for upcycling low‐value poultry by‐products into high‐value flavorful sauce.

## Author Contributions

**Xing Zhang**: conceptualization, methodology, software, data curation, investigation, validation, formal analysis, visualization, project administration, writing – review and editing, writing – original draft. **Sidi Ma**: data curation, formal analysis, writing – review and editing. **Shao‐Quan Liu**: conceptualization, methodology, funding acquisition, supervision, writing – review and editing, project administration.

## Disclosure

All persons who meet authorship criteria are listed as authors, and all authors certify that they have participated sufficiently in the work to take public responsibility for the content, including participation in the concept, design, writing, or revision of the manuscript. Each author certifies that this material or similar material has not been and will not be submitted to or published in any other publication.

## Conflicts of Interest

The authors declare no conflicts of interest.

## Supporting information




**Supplementary Materials**: jfds70564‐sup‐0001‐Appendices.pdf
