# Supplementary material for: Sulfur Supplementation Potentiates the Formation of Meat Aroma Compounds in Thermally Treated Chicken Carcass Hydrolysate
Source: J Food Sci. 2025 Sep 14;90(9):e70564. doi: 10.1111/1750-3841.70564 (PMC12433604; doi:10.1111/1750-3841.70564)
Supplement: Supplementary file 1 — Supplementary Materials: jfds70564‐sup‐0001‐Appendices.pdf [file JFDS-90-0-s001.pdf]

Appendices

Sulfur supplementation potentiates the formation of meat aroma compounds in thermally treated chicken carcass hydrolysate

Xing Zhang [a], Sidi Ma [a], Shao-Quan Liu[a,b] *

[a] Department of Food Science and Technology, National University of Singapore, Science Drive 3, Singapore 117543, Singapore.

[b] National University of Singapore (Suzhou) Research Institute, 377 Lin Quan Street, Suzhou Industrial Park, Jiangsu 215213, China.

*Corresponding author: Shao-Quan Liu, E-mail: fstlsq@nus.edu.sg

Choice of journal:

Journal of Food Science

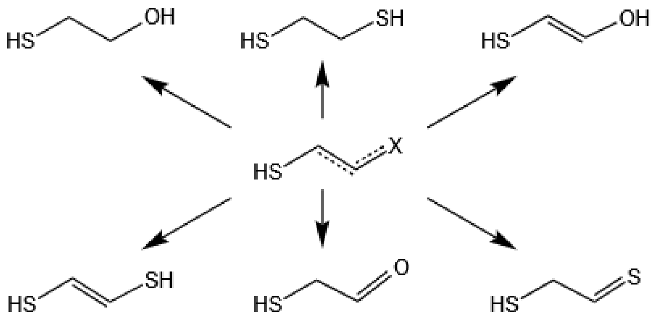

Figure S1. Six structural units of sulfur-containing meaty flavor compounds (X=S/O/SH/OH) adapted from (Sun et al., 2005)

(a) (b)

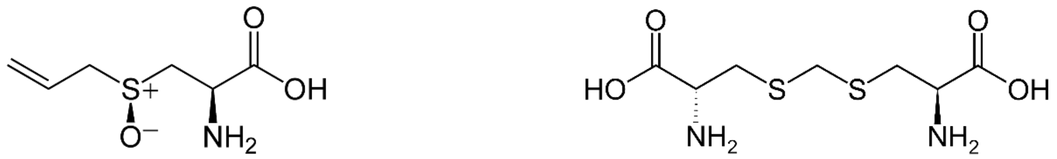

Figure S2. Chemical structures of L-alliins (a) and djenkolic acid (b)

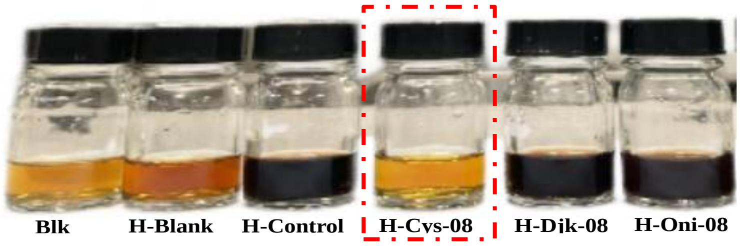

Figure S3. Colour changes of unheated control and heat-treated chicken carcass hydrolysates with S-supplementations (Cys-Blk-08, Oni-Blk-08, Djk-Blk-08, H-Control, H-Cys-08, H-Oni-08, H-Djk-08, refer to Table 1 for detailed descriptions).

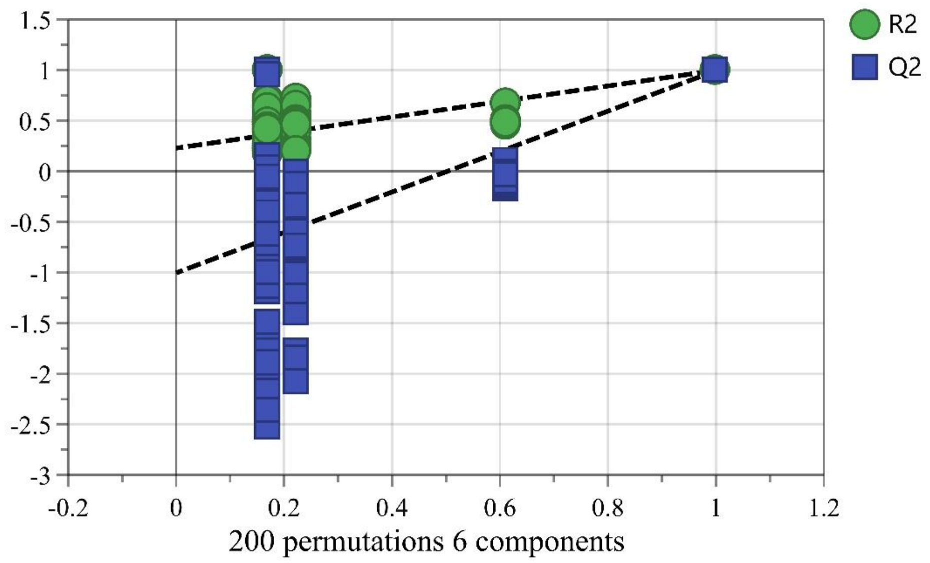

Figure S4. Permutation test results (n = 200) of OPLS-DA mode of GC-MS

Table S1. Volatile compounds by SPME and their concentration in unheated and heated chicken carcass hydrolysates with S-supplementations

No.	Compound identified	LRI	Concentration (ppb)							Odor description
			Unheated control and heated control				Heated samples with different S-supplements			
			Cys-Blk-08	Oni-Blk-08	Djk-Blk-08	H-Control	H-Cys-08	H-Oni-08	H-Djk-08	
	Acids									
1	Acetic acid	1446	-	-	-	-	115.32±5.30	-	-	Sour, pungent,sharp
2	Hexanoic acid	1838	32.57±1.87c	29.85±0.12c	39.42±2.69c	-	172.67±17.24a	10.77±3.19d	77.53±9.21b	Fatty, sour, sweaty
3	Heptanoic acid	1946	-	-	-	-	56.24±1.35a	2.87±0.48	19.28±0.16b	Sour, cheesy, sweat
4	Octanoic acid	2052	-	-	-	-	265.18±10.46a	24.25±3.43c	152.06±2.97b	Cheesy, sweaty, fatty
5	Nonanoic acid	2161	-	-	-	-	175.97±1.52a	8.17±2.18c	44.99±12.01b	Cultured dairy, green
	**Subtotal**		32.57±1.87c	29.85±0.12c	39.42±2.69c		785.31±35.87a	46.06±9.28c	293.86±9.28b	
	Alcohols									
6	1-Pentanol	1251	78.32±1.49	-	-	-	-	-	-	Oil, balsamic, vanilla
7	1-Adamantanol	1293	-	-	-	-	240.84±12.43	-	-	-
8	1-Hexanol	1347	440.15±41.20a	386.89±15.29b	459.05±39.21a	-	-	-	-	Fruity, flower, green
9	1-Octen-3-ol	1434	279.14±12.52a	247.04±22.39b	260.77±2.46b	-	89.01±3.43c	-	-	Fishy, earthy
	**Subtotal**		719.29±53.57	633.93+37.68	719.82±41.67		329.85±15.86			
	Aldehydes									
10	3-Methylbutanal*	-	-	-	-	14.08±1.59b	-	56.24±7.91a	-	Peach, sour, malt, fatty
11	2-Methyl-2-pentenal	1168	-	-	-	-	-	3.32±0.00	-	Fruity
12	Hexanal	1074	632.89±29.39a	206.16±11.51c	572.43±39.48b	142.46±6.81d	76.25±6.94e	23.93±1.35f	141.99±25.37d	Grassy, tallow, fruity

Table S1 *(continued)*

No.	Compound identified	LRI	Concentration (ppb)							Odor description
			Unheated control and heated control				Heated samples with different S-supplements			
			Cys-Blk-08	Oni-Blk-08	Djk-Blk-08	H-Control	H-Cys-08	H-Oni-08	H-Djk-08	
13	2-Heptenal	1325	23.89±1.95a	-	9.69±0.26b	-	-	-	-	Soap, fatty, pungent
14	Nonanal	1379	50.11±4.41a	-	47.75±3.60b	16.97±0.17d	22.53±0.62c	5.65±0.05e	-	Citrus, orange peel
15	2-Furaldehyde	1478	-	-	-	-	64.60±5.79bc	70.69±6.41b	312.80±91.25a	Bready, woody, baked
16	Benzaldehyde	1542	63.64±5.57cd	13.61±1.87e	977.69±53.17a	94.74±2.06c	-	42.55±2.43de	308.87±36.19b	Cherry, almond, burnt
17	Phenylacetaldehyde	1661	-	-	-	-	10.54±0.47b	14.49±1.07b	104.00±11.67a	Honey, green, floral
18	2-Butyloct-2-enal	1678	-	-	-	-	24.26±3.86b	-	95.56±35.36a	Green, fruity, sweaty
19	2-Ethylbenzaldehyde	1698	-	-	-	-	16.29±2.01a	2.64±0.70c	11.29±0.62b	Fruity
20	4-Ethylbenzaldehyde	1736	-	-	-	-	55.73±2.28a	4.16±0.21c	19.19±5.98b	Sweet, almond
21	3,4-Dimethylbenzaldehyde	1840	-	-	-	14.06±0.45c	161.78±25.55a	6.25±0.27c	46.93±16.48b	-
22	2-Methyl-3-(2-furyl) propenal	1918	-	-	-	-	-	2.01±0.34	-	Woody, herbal, spice
23	4-Pentylbenzaldehyde	2041	-	-	-	-	35.88±4.04	-	-	Almond
24	5-Methyl-2-phenyl-2-hexenal	2107	-	-	-	-	-	6.42±1.81b	36.43±2.08a	Butyric, cocoa, fruity
	**Subtotal**		770.53±41.32	219.77±13.38	1607.56±96.51	282.31±11.08	467.86±51.56	238.35±14.64	1077.06±225	
	Ketones									
25	4-Methyl-3-penten-2-one	1132	99.26±1.00b	59.37±1.25d	74.72±2.34c	132.91±3.35a	33.65±2.23e	-	64.97±7.61d	Earthy
26	2-Heptanone	1183	-	-	-	38.54±2.35c	105.28±0.16a	-	81.40±6.26b	Coconut, woody
27	3-Octanone	1246	-	111.00±12.07a	30.65±3.62b	-	-	-	-	Butter, herbal
28	2-Octanone	1276	170.90±19.52a	148.48±16.47b	181.52±9.11a	5.57±0.10d	29.88±1.82c	5.92±0.92d	31.65±0.19c	Earthy, herbal, woody
29	2-Nonanone	1380	21.84±0.56c	33.55±2.34b	22.62±0.51c	-	38.38±3.42a	6.52±2.78d	-	Herbal, fishy, earthy
30	2-Decanone	1490	-	-	-	4.56±0.04d	40.93±1.24b	8.80±2.11c	44.55±1.22a	Floral, orange, peach
31	4-(2-furyl)-2-butanon	1654	-	-	-	-	-	3.28±0.63	-	Caramel, Spicy
32	2-Pentyl-2-cyclopenten-1-one	1763	-	-	-	-	15.29±0.67	-	-	Woody, isojasmone
33	Dihydrojasmone	1878	-	-	-	-	10.90±1.64	-	-	Spice, jasmin, woody
	**Subtotal**		292±21.08	293.03±30.88	234.79±13.24	10.13±0.14	274.31±11.18	24.52±4.58	222.57±15.28	

Table S1 *(continued)*

No.	Compound identified	LRI	Concentration (ppb)							Odor description
			Unheated control and heated control				Heated samples with different S-supplements			
			Cys-Blk-08	Oni-Blk-08	Djk-Blk-08	H-Control	H-Cys-08	H-Oni-08	H-Djk-08	
	Volatile phenols									
34	3-Ethylphenol	1210	-	-	-	-	5.12±0.38	-	-	Musty
35	2,6-Di-tert-butyl-4-methylphenol	1914	-	-	-	-	20.92±1.88	-	-	Vanilla, musty
	**Subtotal**		-	-	-	-	26.04±2.26			
	Sulfur-containing volatiles									
36	3,4-Dimethylthiophene	1256	-	-	-	-	-	8.03±0.12	-	Savory roasted onion
37	2-Methyl-3-furanthiol (MFT)	1307	-	-	-	-	159.62±7.62	-	-	Meaty, roasted
38	2-Methyl-5-(methylthio) furan	1344	-	-	-	-	7.66±0.10	-	-	Mustard, garlic, onion
39	2-Butyl-thiophene	1351	-	-	-	-	6.61±0.43	-	-	Fruity, floral, fried chicken
40	Dipropyl disulfide	1363	-	27.08±0.85	-	-	-	-	-	Burnt, onion, sulfurous
41	2-Furfurylthiol (FFT)	1430	-	-	-	-	213.05±12.75	-	-	Roasted, Sulfurous, meaty
42	2-Pentylthiophene	1451	-	-	-	-	29.52±1.29	-	-	Fatty, fruity, sweet
43	Methional	1488	-	-	-	14.45±1.36	-	-	-	Cabbage, pungent
44	Furfuryl methyl sulfide	1501	-	-	-	-	-	1.54±0.08	57.28±2.52a	Garlic, onion, horseradish
45	Methyl propyl trisulfide	1529	-	-	-	-	-	6.10±0.20	-	Sulfurous, garlic, onion
46	*cis*-1-Propenyl propyl trisulfide	1792	-	94.90±1.35	-	-	-	-	-	-
47	3-Methyl-2-thiophenecarboxaldehyde	1852	-	-	-	-	16.17±4.03	-	-	-
48	2,5-Thiophenedicarboxaldehyde	1986	-	-	-	-	41.19±0.09	-	-	-
49	bis(2-Methyl-3-furyl) disulfide (MFT-MFT)	2177	-	-	-	-	579.76±39.86	-	-	Roasted Meat
50	Furfuryl 2-methyl-3-furyl disulfide	2438	-	-	-	-	25.97±2.12	-	-	Sulfurous, Meaty
51	2-Methyl-3-[2-methyl-3-thienyl) dithio] furan	2494	-	-	-	-	20.72±1.24	-	-	Sulfurous, Meaty
	**Subtotal**			121.98		14.45±1.36	1100.27±29.76	15.67±0.4		

Table S1 *(continued)*

No.	Compound identified	LRI	Concentration (ppb)							Odor description
			Unheated control and heated control				Heated samples with different S-supplements			
			Cys-Blk-08	Oni-Blk-08	Djk-Blk-08	H-Control	H-Cys-08	H-Oni-08	H-Djk-08	
	Others									
52	2-Ethylfuran*		63.50±5.05a	-	16.72±0.05b	-	-	-	-	Earthy, burnt, malty,
53	2-Propylfuran	1022	6.66±0.82	-	-	-	-	-	-	Spicy, fruity, caramellic
54	5-Methyl-3-heptyne	1025	-	-	-	-	50.47±2.28	-	-	-
55	2-Pentylfuran	1206	637.07±7.68b	224.92±39.11b	474.58±32.20b	196.21±1.95b	1416.00±66.80a	214.53±74.64b	1789.48±922.26a	Butter, earthy, beany, green, fruity
56	2-Hexylfuran	1324	-	-	-	-	56.30±3.45a	6.25±1.85b	-	
57	1,3-Di-tert-butylbenzene	1340	-	-	-	6.71±0.35	-	-	-	
58	1,3,5-Undecatriene	1399	-	-	-	-	-	-	12.74±2.07	Green
59	3-Phenylfuran	1881	-	-	-	-	32.44±0.46b	27.15±8.60b	161.89±14.20a	
60	4-Hydroxy-5-methyl-3-furanone	2144					71.57±11.49a	5.93±1.42c	20.85±4.21b	Bready, caramel
	**Subtotal**		707.23±13.55	224.92±39.11	491.30±32.25	202.92±2.3	84.48	253.86±86.51	1984.96±942.74	

Values are expressed as the mean of triplicate independent experiments ± SD.

LRI, linear retention index, determined on a DB-FFAP column relative to the n-alkanes of C10-C35.

“-” : Compound not detected.

*: Compound identified by MS method only.

Different lowercase letters in a row indicate statistical differences (*P* <0.05) between differentially fermented chicken hydrolysates.

Odor descriptions were adopted from Flavor DB database (https://cosylab.iiitd.edu.in/flavordb/), Chemical Book database (https://www.chemicalbook.com/), PubChem database (https://pubchem.ncbi.nlm.nih.gov/), and , and The Good Scents Company (https://www.thegoodscentscompany.com/).

Table S2. Qualitative detection of volatiles in unheated and heated chicken carcass hydrolysates with S-supplementations using GC-IMS

Count	Compound	CAS#	Formula	MW	RI[1]	RT[2]	DT[3]
1	Acetone	C67641	C_3H_6O	58.1	844	231.387	1.12362
2	Ethanol	C64175	C_2H_6O	46.1	929.5	265.834	1.14317
3	2-Butanone	C78933	C_4H_8O	72.1	900.8	253.763	1.25702
4	Acetic acid ethyl ester	C141786	$C_4H_8O_2$	88.1	881.2	245.814	1.34804
5	Methyl propanoate	C554121	$C_4H_8O_2$	88.1	916.4	260.24	1.33417
6	2-Methyl propanal	C78842	C_4H_8O	72.1	816.7	221.377	1.28677
7	Ethyl formate	C109944	$C_3H_6O_2$	74.1	808.5	218.433	1.07638
8	Propanal	C123386	C_3H_6O	58.1	775.2	206.95	1.14228
9	Triethylamine	C121448	$C_6H_{15}N$	101.2	730.7	192.524	1.08679
10	1-Propanol (monomer)	C71238	C_3H_8O	60.1	1040.3	327.663	1.11222
11	1-Propanol (dimer)	C71238	C_3H_8O	60.1	1040.3	327.663	1.24747
12	Propanethiol	C107039	C_3H_8S	76.2	830.5	226.382	1.37116
13	Tetrahydrofuran	C109999	C_4H_8O	72.1	879	244.931	1.23475
14	Butanal	C123728	C_4H_8O	72.1	890.8	249.641	1.30065
15	2-Methylbutanal	C96173	$C_5H_{10}O$	86.1	934.3	267.895	1.3885
16	3-Methylbutanal	C590863	$C_5H_{10}O$	86.1	919.2	261.418	1.41971
17	Propyl acetate	C109604	$C_5H_{10}O_2$	102.1	976.1	286.738	1.4752
18	2-Pentanone	C107879	$C_5H_{10}O$	86.1	985.5	291.155	1.38272
19	n-Pentanal	C110623	$C_5H_{10}O$	86.1	987.4	292.038	1.43012
20	2-Methylbutanoic acid, methyl ester	C868575	$C_6H_{12}O_2$	116.2	1013.1	307.937	1.54918
21	2-Butanol (monomer)	C78922	$C_4H_{10}O$	74.1	1031.5	321.186	1.15152
22	2-Butanol (dimer)	C78922	$C_4H_{10}O$	74.1	1027.5	318.241	1.32261
23	2-Methyl butanoic acid ethyl ester (monomer)	C7452791	$C_7H_{14}O_2$	130.2	1065.5	347.095	1.23707
24	2-Methylbutanoic acid ethyl ester (dimer)	C7452791	$C_7H_{14}O_2$	130.2	1054.2	338.262	1.65669
25	1-Penten-3-ol	C616251	$C_5H_{10}O$	86.1	1164.8	458.111	0.94483
26	2-Heptanone (monomer)	C110430	$C_7H_{14}O$	114.2	1186.3	488.809	1.26114
27	2-Heptanone (dimer)	C110430	$C_7H_{14}O$	114.2	1186	488.405	1.64148
28	2-Pentylfuran	C3777693	$C_9H_{14}O$	138.2	1237.6	566.767	1.25986

Table S2 *(continued)*

Count	Compound	CAS#	Formula	MW	RI[1]	RT[2]	DT[3]
29	1-Pentanol (monomer)	C71410	$C_5H_{12}O$	88.1	1261.5	606.824	1.25451
30	1-Pentanol (dimer)	C71410	$C_5H_{12}O$	88.1	1261.5	606.824	1.50964
31	1-Pentanol (polymer)	C71410	$C_5H_{12}O$	88.1	1262.6	608.854	1.82892
32	3-Hydroxy-2-butanone (monomer)	C513860	$C_4H_8O_2$	88.1	1293.5	665.023	1.05906
33	3-Hydroxy-2-butanone (dimer)	C513860	$C_4H_8O_2$	88.1	1293.1	664.346	1.33657
34	Benzaldehyde (monomer)	C100527	C_7H_6O	106.1	1527	1056.902	1.15371
35	Benzaldehyde (dimer)	C100527	C_7H_6O	106.1	1525.6	1053.968	1.48032
36	Acetic acid (monomer)	C64197	$C_2H_4O_2$	60.1	1482.3	967.426	1.05155
37	Acetic acid (dimer)	C64197	$C_2H_4O_2$	60.1	1481.5	965.959	1.15216
38	1-Octen-3-ol	C3391864	$C_8H_{16}O$	128.2	1470.7	945.423	1.16919
39	Nonanal (monomer)	C124196	$C_9H_{18}O$	142.2	1401	823.677	1.47723
40	Nonanal (dimer)	C124196	$C_9H_{18}O$	142.2	1398.3	819.277	1.95708
41	1-Hexanol (monomer)	C111273	$C_6H_{14}O$	102.2	1367.5	770.871	1.32708
42	1-Hexanol (dimer)	C111273	$C_6H_{14}O$	102.2	1366.5	769.405	1.64285
43	Butyl hexanoate	C626824	$C_{10}H_{20}O_2$	172.3	1429.8	872.082	1.4633
44	Methyl benzoate	C93583	$C_8H_8O_2$	136.1	1593.3	1205.051	1.20789
45	(*Z*)-2-Penten-1-ol	C1576950	$C_5H_{10}O$	86.1	1332.1	718.74	0.95274
46	2-Hexanone (monomer)	C591786	$C_6H_{12}O$	100.2	1085.7	363.459	1.20172
47	2-Hexanone (dimer)	C591786	$C_6H_{12}O$	100.2	1089.4	366.56	1.508
48	1-Hexanal	C66251	$C_6H_{12}O$	100.2	1092.4	369.144	1.57847
49	2-Methyl-1-propanol	C78831	$C_4H_{10}O$	74.1	1099.4	375.766	1.17016
50	1-Butanol (monomer)	C71363	$C_4H_{10}O$	74.1	1147.5	434.685	1.18022
51	1-Butanol (dimer)	C71363	$C_4H_{10}O$	74.1	1148.2	435.63	1.38049
52	Heptanal	C111717	$C_7H_{14}O$	114.2	1190	494.338	1.33146
53	3-Methyl-2-butenal	C107868	C_5H_8O	84.1	1206.6	518.597	1.0936
54	Isoamyl butyrate	C2050013	$C_9H_{18}O_2$	158.2	1208.2	520.985	1.37245

Table S2 *(continued)*

Count	Compound	CAS#	Formula	MW	RI[1]	RT[2]	DT[3]
55	3-Methylbutan-1-ol (monomer)	C123513	$C_5H_{12}O$	88.1	1214.5	530.537	1.24243
56	3-Methylbutan-1-ol (dimer)	C123513	$C_5H_{12}O$	88.1	1213.8	529.343	1.49151
57	Ethyl hexanoate	C123660	$C_8H_{16}O_2$	144.2	1244.3	577.7	1.33172
58	1-Hydroxy-2-propanone (monomer)	C116096	$C_3H_6O_2$	74.1	1310.1	688.147	1.03407
59	1-Hydroxy-2-propanone (dimer)	C116096	$C_3H_6O_2$	74.1	1308.8	686.356	1.23773
60	(*E*)-2-Heptenal (monomer)	C18829555	$C_7H_{12}O$	112.2	1328.2	713.221	1.26279
61	(*E*)-2-Heptenal (dimer)	C18829555	$C_7H_{12}O$	112.2	1328.2	713.221	1.68577
62	5-Nonanone	C502567	$C_9H_{18}O$	142.2	1323.5	706.654	1.35209
63	3-Nonanone	C925780	$C_9H_{18}O$	142.2	1338.2	727.549	1.36775
64	2-Octanone (monomer)	C111137	$C_8H_{16}O$	128.2	1271.4	624.267	1.32545
65	2-Octanone (dimer)	C111137	$C_8H_{16}O$	128.2	1271.1	623.67	1.7594
66	3-(Methylthio)propanal (monomer)	C3268493	C_4H_8OS	104.2	1463.9	932.881	1.10009
67	3-(Methylthio)propanal (dimer)	C3268493	C_4H_8OS	104.2	1466.1	936.856	1.41427

Note: The suffixes monomer and dimer respectively refer to the monomer and dimer of the same substance, and the numbers represent unidentified peaks.

CAS#: the registration number of chemical substances by *Chemical Abstracts Service*.

[1] Represents the retention index calculated on DB-WAX capillary column using n-alkanes C4 -C9 as external standards.

[2] Represents the retention time in the capillary GC column.

[3] Represents the drift time in the drift tube.
